# Pathobiont release from dysbiotic gut microbiota biofilms in intestinal inflammatory diseases: a role for iron?

**DOI:** 10.1186/s12929-018-0495-4

**Published:** 2019-01-03

**Authors:** Andre Gerald Buret, Jean-Paul Motta, Thibault Allain, Jose Ferraz, John Lawrence Wallace

**Affiliations:** 1 Departments of Biological Sciences, and Pharmacology and Therapeutics, Inflammation Research Network, University of Calgary, 2500 University Dr. N.W, Calgary, T2N 1N4 Canada; 2 Institute of Digestive Health Research, INSERM UMR1220, Université Toulouse Paul Sabatier, Toulouse, France; 3 Division of Gastroenterology, Cumming School of Medicine, University of Calgary, Calgary, T2N 1N4 Canada

**Keywords:** Microbiota biofilm, Microbiome, Pathobionts, Giardia, Campylobacter, Post-infectious IBS, Inflammatory bowel disease, Crohn’s disease, Colo-rectal cancer, Iron, Dysbiosis, Metabolome, Enteropathogen, Mucus

## Abstract

Gut microbiota interacting with an intact mucosal surface are key to the maintenance of homeostasis and health. This review discusses the current state of knowledge of the biofilm mode of growth of these microbiota communities, and how in turn their disruptions may cause disease. Beyond alterations of relative microbial abundance and diversity, the aim of the review is to focus on the disruptions of the microbiota biofilm structure and function, the dispersion of commensal bacteria, and the mechanisms whereby these dispersed commensals may become pathobionts. Recent findings have linked iron acquisition to the expression of virulence factors in gut commensals that have become pathobionts. Causal studies are emerging, and mechanisms common to enteropathogen-induced disruptions, as well as those reported for Inflammatory Bowel Disease and colo-rectal cancer are used as examples to illustrate the great translational potential of such research. These new observations shed new light on our attempts to develop new therapies that are able to protect and restore gut microbiota homeostasis in the many disease conditions that have been linked to microbiota dysbiosis.

The gut microbiome is made of the aggregate collection of genomes and genes contained in its commensal viruses, bacteria, fungi, and Eukarya. Together, these microorganisms are known as the gut microbiota, although at times, the term microbiota is used to specifically refer to the bacterial communities within this complex consortium. The most recent estimates suggest that the ratio of gut bacterial cells to human body cells is approximately 1 to 1 [1]. Gut microbiota live in close association with themselves and with the outer layer of the host mucus, as intricate biofilm communities and free-swimming bacteria that disperse from them. The symbiotic relationship between the host and its gut microbiota begins at birth, and is critical to the overall evolutionary fitness and health of the host [2–4]. Both exogenous as well as endogenous environmental factors are able to modify gut microbiota, and these alterations may in turn lead to detrimental health effects. After elaborating on the biofilm mode of growth of gut microbiota, the present review will focus on how disruptions (“dysbiosis”) within these microbial communities may contribute to enteropathogen-induced disorders, to Inflammatory Bowel Diseases, or to colo-rectal cancer. Microbiota dysbiosis has indeed been linked to a variety of diseases in the gastrointestinal tract as well as in other organs, including the joints, the skin, the eyes, the vasculature, the lungs, and even the central nervous system [4–7]. Much needed causal studies on these relationships are found at increasing rates in the scientific literature. Together, these observations indicate that gut commensal microorganisms may become pathobionts. In humans, animals and plants, pathobionts and opportunistic pathogens are known as temporarily benign microbes or commensals that under environmental or host pressure may cause disease [8]. As much as one third of a host’s metabolome circulating in its blood comes from a bacterial origin, which sheds light on the great potential for gut microbial commensals and pathobionts to shape homeostatic functions throughout the body [9]. While the mechanisms remain largely undefined, this review will present current research findings that are starting to uncover some of these processes.

## Background

The well accepted plasticity of the microbiota begs the question of whether the identities of present-day human gut microbial communities originate through inheritance or from environmental pressures. Ground-breaking comparative studies in wild apes, and in humans from Africa or from the Northern hemisphere clearly demonstrate that some of the major families of the gut microbiota have been evolving from common ancestors for more than 15 million years [10]. Since the different species of apes evolved separately from their ancestors, their gut microbiota also diverged and coevolved in parallel, adapting to various environmental factors including diet, gastrointestinal disorders, and habitat, which may in turn have contributed to the significant phyla shifts observed between human gut microbiota of the industrialized World versus those in low income countries [10, 11]. The plasticity of the gut microbiota is reflected in the significant microbiota dysbiosis observed during a variety of disorders. Hence, environmental factors dominate over genetics in determining gut microbiota, which suggests that similar therapeutic approaches aimed at shaping gut microbiota may be applied across different genetic backgrounds. The explosive growth of data on the assembly and stability of gut microbiota, and on how in turn these may control health and disease is both captivating and daunting. In an attempt to reveal common pathways, this review will focus on how disruptions of microbiota biofilms may lead to post-infectious enteritis disorders, to Inflammatory Bowel Diseases (IBD), or to colo-rectal cancer. It has become evident that these emerging views have the potential to revolutionize the development of future therapeutics.

### Gut microbiota biofilms

**Fig. 1:**
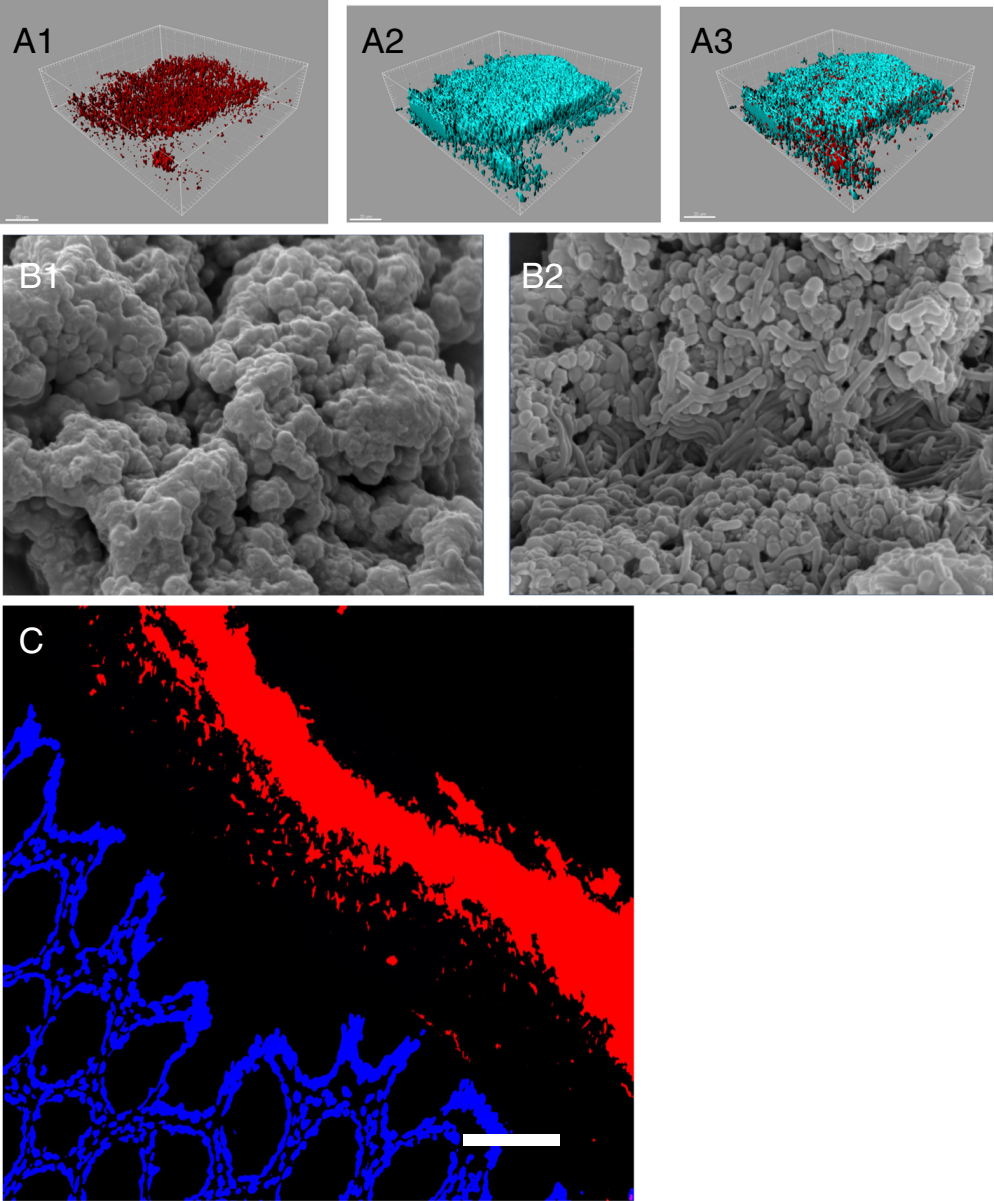
Gut Microbiota live as biofilms: a) Confocal laser micrograph of microbiota grown form a healthy human donor colonic biopsy ex vivo on the Calgary Biofilm Device ™, and illustrating their biofilm mode of growth (A3, merge image). The microbiota visibly contain a thick exopolysaccharide coating typical of bacterial biofilms (A2, wheat germ agglutinin stain) covering live bacteria (A1). Bars = 20 μm. B) Human microbiota biofilms grown on the Calgary Biofilm Device ™ and observed under scanning electron microscopy. The slimy exopolysaccharide coating of the biofilm hides underlying bacterial morphology in healthy conditions (B1), and this exopolysaccharide can be lost upon exposure to an enteropathogen like *Giardia* sp. (B2). C) Gut microbiota in the colon of a healthy rat, illustrating the biofilm sheet formed by the commensals (red), separated from the epithelial surface (blue) by the intestinal mucus barrier (not stained). Bar = 50 μm.

**Fig. 2:**
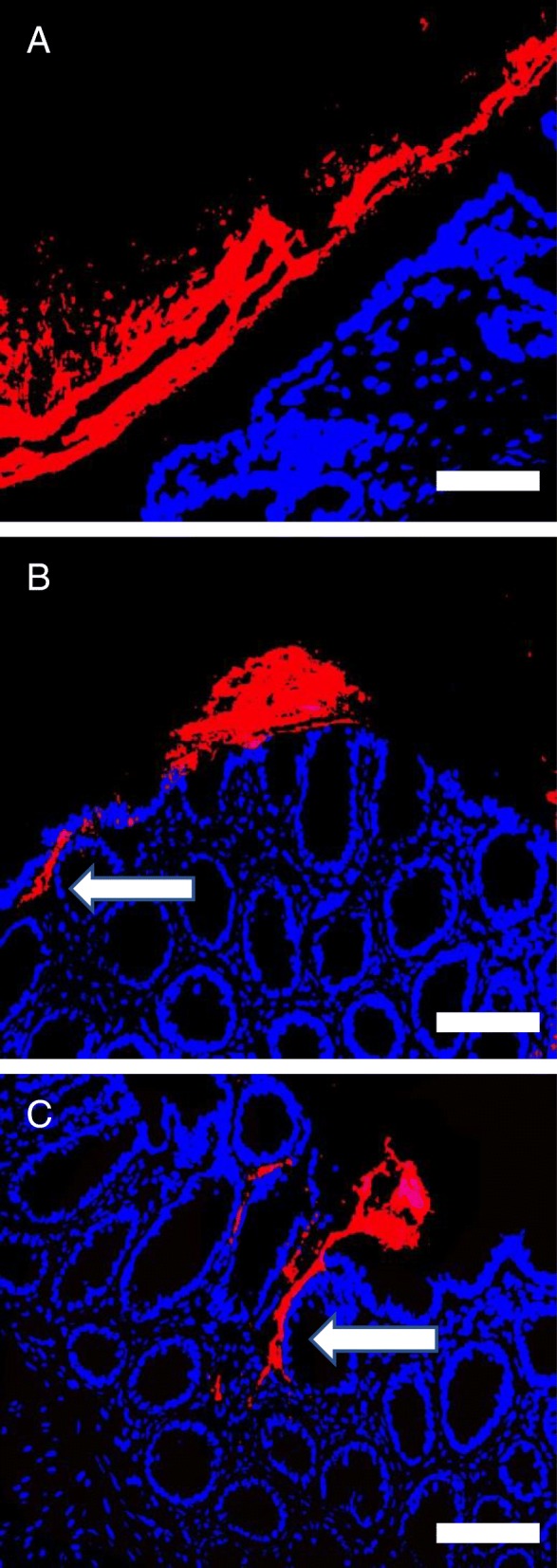
Dysbiotic microbiota (red) in rats with experimental colitis induced by DNBS (B and C) compared to control non-inflamed tissue (**a**). Fragments of the dysbiotic microbiota biofilm (**b**,**c**, in red) directly adhere to the epithelial surface (blue), and releases invasive pahobionts seen in the process of translocation (arrows). Bars = 50 μm. (Modified from reference 20)

**Fig. 3:**
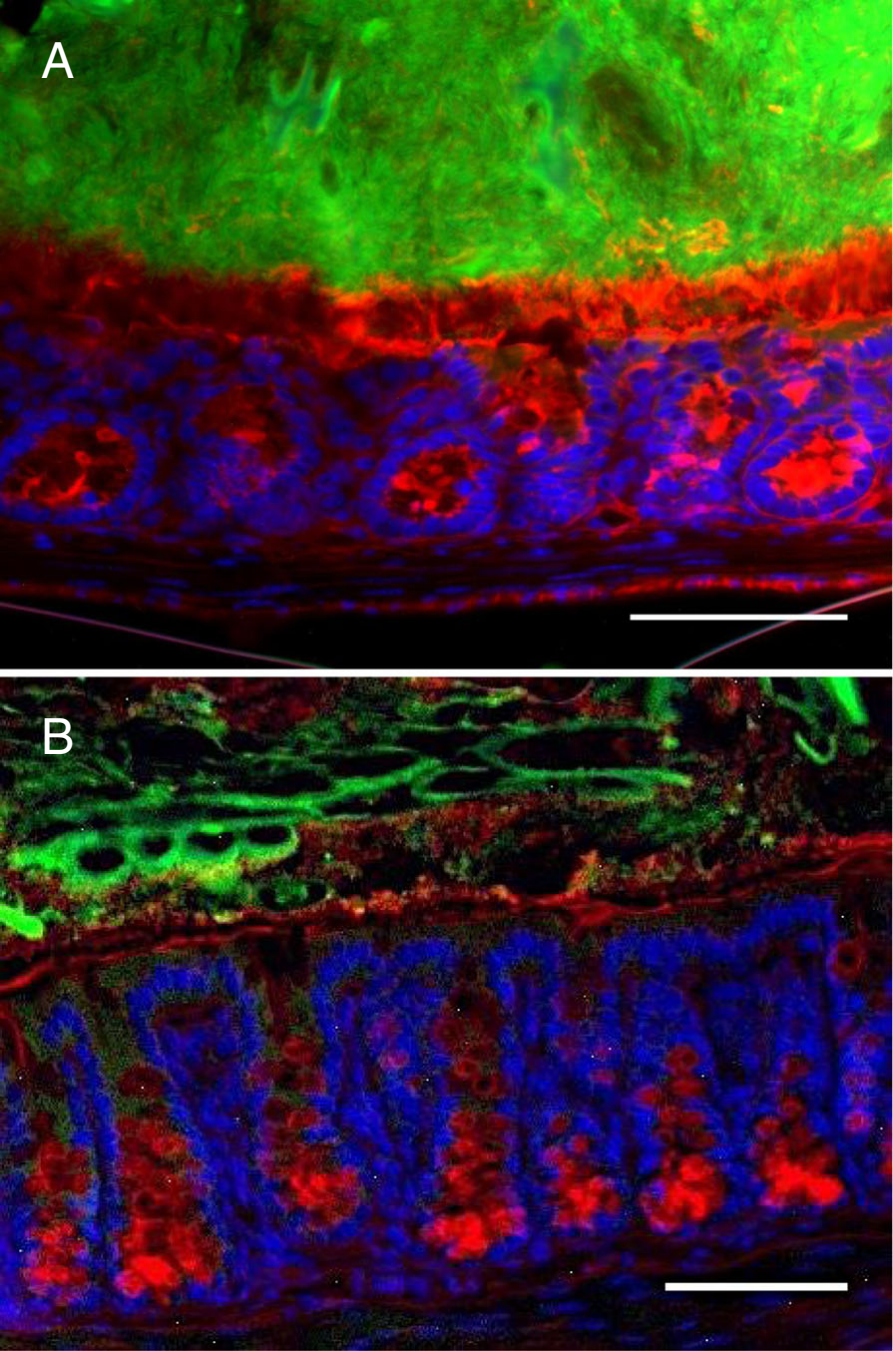
Enteropathogen-induced abnormalities of the colonic microbiota biofilm phenotype (green) is associated with disruption of the mucus barrier (red) in mice infected with *Giardia* sp. for 7 days (**b**), compared to control tissue (**a**). This provides researchers with a powerful model to investigate the mechanisms and consequences of gut microbiota biofilm disruptions, and subsequent invasion of pathobionts. Bars = 200 μm). (Modified from reference 36)

**Fig. 4:**
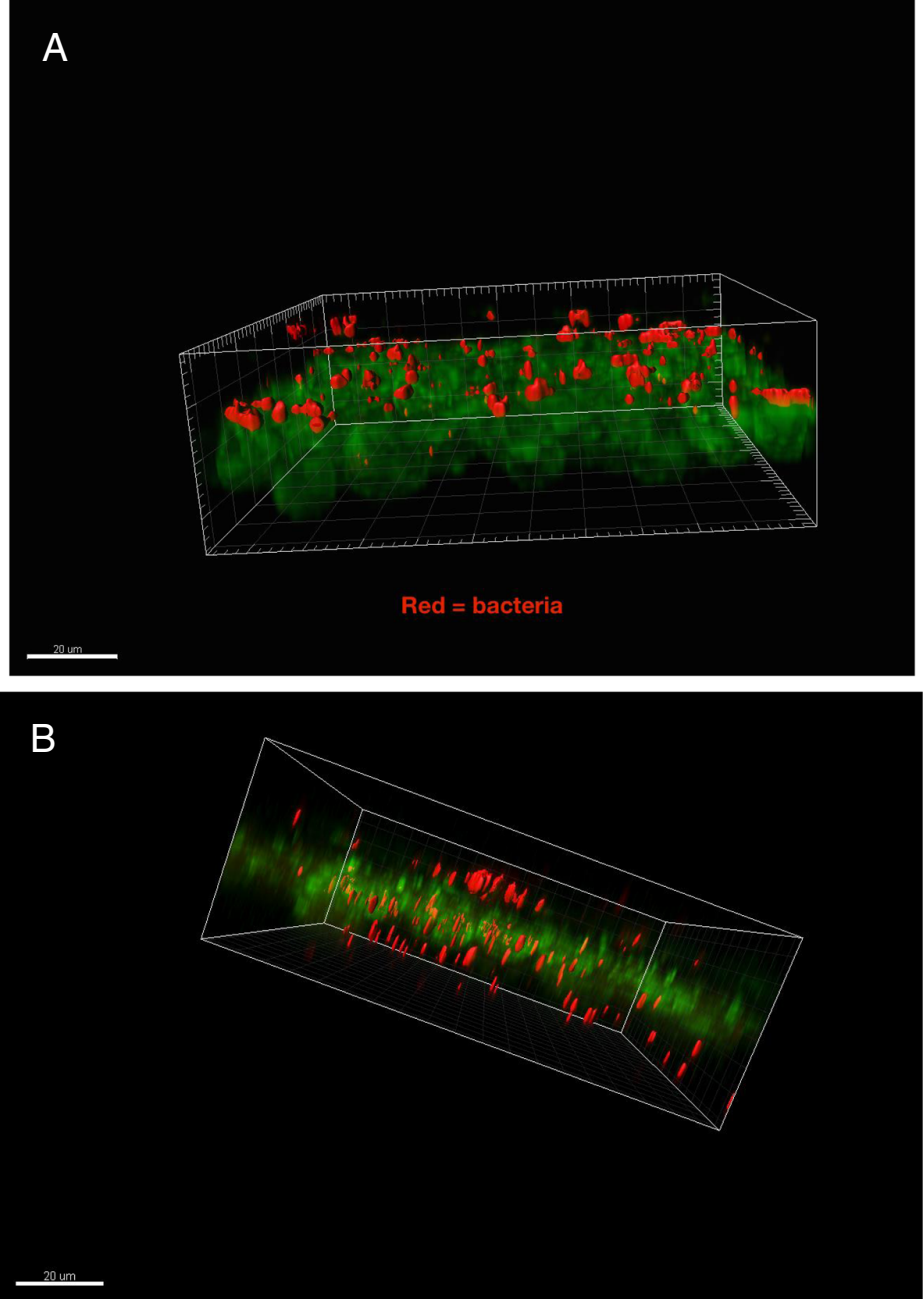
Confical laser micrographs of human microbiota (red) grown on the Calgary Biofilm Device and then incubated with human intestinal epithelial cells (green). Biofilm bacteria dispersed from microbiota of donors with Crohn’s Disease readily translocate (arrows) across the monolayers (**b**) whereas bacteria from microbiota of healthy donors do not (**a**). Bars = 20 μm)

The complex poly-microbial communities of the gut microbiota reside over the intestinal mucus as exopolysaccharide-coated biofilms, that disperse planktonic (free-swimming) bacteria, as they do elsewhere in nature [12–16]. The biofilm mode of growth allows to retain water, to protect against antimicrobial substances and enzymes, and facilitates quorum sensing and horizontal gene transfer [14, 15]. The biofilm mode of growth in gut microbiota seems to be conserved as the layers of bacteria in the honey bee’s ileum are enriched with genes linked to biofilm formation, encoding for type IV pili, motility, flagella, intracellular trafficking, RTX (Repeats-in-Toxins) adhesins, and biofilm-associated proteins [17]. In disease, combined with disruptions of microbiota biofilm phenotypes and altered metabolomics, planktonic bacteria dispersed from the biofilm communities may become pathobionts in the gut (Table 1) [16, 18–27]. In other words, disease may be triggered by the same bacterial species that colonizes healthy individuals. Recent findings offer new insights into how a pro-inflammatory T-cell -mediated response could be restrained by homeostatic commensals, or conversely, triggered by pathobionts. Indeed, in the case of intestinal inflammation driven by *Helicobacter pylori*, this process was found to be regulated by a pathobiont-specific peripherally derived regulatory T cell population called pT_reg_ [28]. Moreover, it appears that experimental administration of pathobionts may synergize with commensal microbiota to exacerbate pathology, shedding new light on the complexity of how pathobionts may cause disease [29]. The biological significance of the close interactions between gut microbiota biofilms and host mucus is further underscored by the observation that intestinal biofilm bacteria living on mucin differ metabolically and phylogenetically from those living in a planktonic, motile state [30]. Taken together, these observations underscore the importance of characterizing phenotypic and functional alterations of the microbiota biofilms, beyond the routine identifications of relative bacterial abundance and diversity. Moreover, the human gut mucosal microbiota, which interact with the host, differ from the fecal microbiome [31, 32]. More research is needed to characterize mucosal, rather than fecal, microbiota throughout the entire length of the gastrointestinal tract, and their role in health and disease. Recent findings have revealed that colonic mucosal microbiota obtained from human biopsies may be grown as biofilms ex vivo allowing for mechanisitic studies (Fig. 1) [26]. In rodent models of experimental colitis, microbiota biofilms are fragmented, adhere to epithelial cells, and release invasive bacterial pathobionts [20] (Figs. 1 and 2). In patients with IBD, gut microbiota biofilm clusters can also be seen adhering tightly to the epithelial surface, obviously having bypassed the mucus barrier [33]. Bacteria dispersed from microbiota biofilms grown from biopsy tissues of patients with Crohn’s Disease or Ulcerative Colitis are able to invade intestinal epithelia and potentiate pro-inflammatory signals (Fig. 2) [26]. These phenomena have been implicated in disease pathogenesis [20, 23, 26, 33–35]. Enteropathogen-induced pathogenic microbiota biofilm alterations and pathobiont dispersion coincide with mucus disruption (Figs. 2 and 3) [18, 36]. Bacteria dispersed from human microbiota biofilms obtained from patients with IBD (Fig. 4), or from microbiota made dysbiotic by exposure to the intestinal prozoan parasite *Giardia* sp. are able to translocate epithelial barriers, and to induce the production of pro-inflammatory CXCL-8 in human epithelia and in germ-free mice [18, 26]. Similar adherence of microbiota biofilm fragments to the epithelial surface has recently been reported in polyposis and colorectal cancer, where secretion of biofilm metabolites such as polyamines have been detected at concentrations 62-times higher than in microbiota biofilms from healthy tissues [37–41]. These observations highlight the importance of identifying mucosal microbiota biofilm metabolomic characteristics in disease pathogenesis [26, 39, 42, 43]. Beyond abnormalities in their taxonomic representations, a better understanding of how phenotypic and functional disruptions of commensal gut mucosal biofilm communities are regulated will pave the way towards novel therapies. Investigations of biofilms in mono-and multi-species communities will shed new light on our understanding of microbial metabolism, genetic variability, antibiotic resistance, and of mechanisms leading to post-transcriptional modifications.

**Table 1:** Key concepts

*Microbial Biofilms*: Aggregates of microbial communities (best characterized for bacteria) that adhere to a surface and to each other, embedded within an extra-cellular matrix made of polysaccharides, proteins, and extracellular DNA. This represents the most common mode of growth of bacteria in nature.	
*Planktonic bacteria*: Bacteria living in single form, swimming or floating in their environment	
*Pathobionts*: Temporarily benign microbes, or commensals, that under environmental or host pressure may cause disease.	
*Gut microbiome*: The aggregate collection of genomes and genes found in the gut microbiota.	
*Gut microbiota*: The polymicrobial communities of viruses, bacteria, Archea, and Eukarya living as commensals in the gut.	
*Dysbiosis*: Structural and /or functional imbalance of the gut microbiota.

### Enteropathogen-induced disruptions of microbiota biofilms

In addition to shaping host immunity and gut homeostasis at birth, and to the digestion of undigested nutrients, one of the main functions of the gut microbiota is to protect the host against invading pathogens and against the overgrowth of pathobionts [2, 4]. While these effects are best characterized for bacterial pathogens, they also operate against parasitic and viral pathogens [44–51]. The mechanisms include selective colonization sites, competitive niche exclusion via metabolic interactions and steric hindrance, production of antimicrobials by commensals (eg. bacteriocin), control of pathogen replication, modulation of the mucus barrier, as well as the induction and/or inhibition of specific host immune responses [26, 28, 36, 44, 46, 49–51]. Conversely, much less is known of how enteropathogens are able to affect commensal microbiota. Based on the observations that enteric infections are often followed by post-infectious bouts of Irritable Bowel syndrome, causing flares in patients with IBD, and leading to a variety extra-intestinal complications, recent studies have investigated whether a common pathway whereby these effects may arise is through pathogen-induced disruptions of the gut microbiota, which in turn may drive pathology even when the instigating micro-organism has been cleared [5, 16, 27, 52, 53].

Populations in industrialized countries, with their characteristic high protein and high fat diets, harbour different microbiota than those living in rural areas of developing countries, where a polysaccharide-rich diet is the norm [54, 55]. In rural areas of low income countries, there is an increased representation of mostly gram-negative Bacteroidetes, which can hydrolyze undigestable xylose fibers, whereas gram-positive Firmicutes are the predominant bacterial phylum in high income countries [54, 55]. Both phyla account for more than 95% of the bacteria present in the human gut [4, 5, 7, 11]. The relative sensitivity of these microbiota constituents to enteropathogens remains poorly understood. Recent research from our laboratory and others have now clearly demonstrated that exposure to acute enteropathogens represents yet another important environmental factor able to shape the gut microbiota. The pathogens found to modify human and animal microbiota include bacteria, parasites (Protozoa and Helminths), and viruses (the potential role of Archea is not discussed in this review) [18, 45–51, 56–61]. The mechanisms implicate the release of pathogenic products that may affect both microbiota and host components, modifications of the mucus barrier, redistribution of epithelial Toll-Like Receptors, as well as modulation of host immune responses as least in part by promoting regulatory Tcells that suppress protective responses to inflammatory stimuli [18, 36, 50, 57, 59, 62]. Enteropathogen-modified microbiota directly affect host immunity, and indeed these dysbiotic microbiota are able to cause or exacerbate gut inflammation [18, 19, 36, 45, 59, 62]. Intriguingly, even remote infections, such as respiratory infections with influenza virus, are able to cause gut microbiota dysbiosis [58]. Recent reports also started to shed light on how enteropathogens may modify gut microbiota biofilm phenotype and function [27]. The findings indicate that the Protozoan parasite *G. duodenalis* directly alters relative bacterial abundance and beta diversity of human microbiota biofilms, most strikingly by increasing the representation of *Clostridiales* bacteria (belonging to the Firmicutes phylm) [18]. The parasite also disrupts the biofilm exopolysaccharide and promotes the release of pathobionts; these in turn are able to translocate through human epithelia, as well as in germ-free mice, where they induce the production of pro-inflammatory mediators like CXCL-8 and IL-1 [18]. These microbiota alterations do not occur upon exposure to the commensal bacterium *Escherichia coli* [18]. In contrast, the enteropathogen *Campylobacter jejuni* is able to modify gut microbiota [61], and to promote the expression of latent virulent genes in non-invasive *E. coli*, including fimbrial genes (*fimA*, *sfmF)*, flagellar genes (*fliD)*, and genes regulating Hemolysin E (*hlyE*); these effects are associated with disruptions of TLR4 gene expression, and promote the release of pro-inflammatory CXCL-8 in human intestinal epithelial cells [19]. Studies also found that exposure to *C. jejuni* promotes *E. coli* adherence to, and subsequent translocation through, intestinal epithelial cells [19, 62]. Translocation is facilitated by hijacking the host lipid raft pathway as well as via epithelial M-cells [62–64]. Other studies demonstrated that infection with *C. jejuni* indeed exacerbates post-infectious murine colitis upon a mild Dextran Sulfate Sodium (DSS) challenge [65]. Consistent with an enteropathogen-induced dispersion of pathobionts, the transient infection promoted the translocation of commensal bacteria to the spleen and liver, depolarized epithelial TLR9, and worsened post-infectious DSS colitis [65]. These observations shed light on how infections with *C. jejuni*, and other enteropathogens, including parasites and viruses, may exacerbate inflammation in patients with IBD, lead to post-infectious Irritable Bowel Syndrome, and perhaps contribute to extra-intestinal complications, such as haemolytic-uremic syndrome, endocarditis, and a variety of others [52, 66–71]. Recent studies found that human microbiota rendered dysbiotic by exposure to enteropathogens, of when obtained from patients with IBD, in association with their elevated content of activated virulence genes, cause lethal toxicity in the nematode *Caenorhabditis elegans* [26, 72]. In situ examination of biopsies from patients with IBD revealed the increased uptake of non-invasive, commensal, *E. coli* via the follicle-associated epithelial M cells [73], the same phenomenon found to be facilitated by *C. jejuni* [64]. In IBD, invading commensal *E. coli* were shown to co-localize with dendritic cells, which correlated with increased levels of the pro-inflammatory cytokine TNF-α [73]. Finally, noteworthy to this discussion, enteric murine norovirus can add to the function of commensal bacteria and act as a beneficial commensal, in a type I interferon signaling-dependent fashion [57]. While the mechanisms require further investigation, these findings indicate that eukaryotic viruses of the microbiota are capable to support intestinal homeostasis, as do bacterial commensals. This effect goes well beyond the known effects of bacteriophages on commensal microbiota. Viral participation in the microbiota biofilm mode of growth remains obscure. Finally, in addition to direct effects on microbiota through secreted products, enteropathogens may also modulate colonization by infectious or commensal bacteria in the gut by inducing the production of anti-microbial peptides from the host epithelium, and/or by modulating the host inflammatory response; in turn, these effects may attenuate or exacerbate disease symptoms during enteric infection [74–79]. These observations further underscore the significance of microbial-microbial interactions to gut homeostasis.

In summary, exposure to enteropathogens shapes the gut microbiota, throughout life. Importantly in the context of this review, beyond modifying relative bacterial abundance and diversity and modulating host innate immunity, enteropathogens directly modify the phenotype of the gut microbiota biofilm, induce its adherence to the epithelium by allowing it to bypass the mucus barrier, activate latent virulence genes in commensal bacteria, and promote the release of pathobionts which are able to induce and exacerbate intestinal inflammation, in the absence of the instigating pathogen (Figs. 1, 2 and 3). These may contribute to the common occurrence of intestinal and extra-intestinal complications following enteric infections.

### Gut microbiota biofilm disruptions in inflammatory bowel diseases and Colo-rectal cancer

Gut microbiota dysbiosis plays a key role in the pathogenesis of Inflammatory Bowel Diseases as well as in the development of colo-rectal cancer and patients’ responses to cancer immunotherapy, but the mechanisms remain incompletely understood. Mice with experimental colitis as well as human patients with colo-rectal cancer contain more bacteria with carcinogenic capabilities. These include colibactin-producing pathobiont strains derived from commensal *E. coli* as well as other pathobionts, as discussed below [80]. Colibactin acts as a genotoxin that establishes DNA interstrand cross-links in epithelial cells [81]. These studies and others have established a link between inflammatory diseases of the gut and the development of colo-rectal cancer via disruptions of the gut microbiota, adding to the commonly accepted role of inflammatory mediators as carcinogenic DNA damage inducers in gut epithelial cells [82–84]. Alterations in bacterial abundance and diversity in IBD and colo-rectal cancer have been well characterized, and are reviewed elsewhere [4, 5, 82, 85, 86]. In an attempt to identify new therapeutic targets based on shared mechanisms, this discussion will focus instead on the recently proposed role of phenotypic and functional microbiota biofilm alterations in disease pathogenesis [18, 26, 27, 33, 40, 87].

#### Mucin depletion and microbiota adherence

Degradation of mucosal extracellular matrix components by bacterial-derived metalloproteinases has been suggested to contribute to the pathogenesis of IBD [88]. Whether this bacterial proteolytic activity is also able to alter the exopolysaccharide coat of microbiota biofilms is unclear. We recently demonstrated that the disruptions of microbiota biofilm exopolysaccharides induced by *Giardia* were triggered at least in part by the pathogen’s cathepsin proteases [18]. These same microbial proteases also exhibit mucinase activity [36]. Mucin depletion is a well-established characteristic of colitis [89], and represents a preneoplastic lesion in colo-rectal cancer [90]. In patients with IBD, as well as in experimental models of colitis (Fig. 2), gut microbiota biofilm fragments have been observed adhering tightly to the epithelial surface, obviously having bypassed the mucus barrier [33, 35]. Similarly, adherent microbiota biofilm fragments stick to the epithelial surface in polyposis and colorectal cancer [40]. In all instances, this modified biofilm phenotype has been linked to disease development.

#### Healthy microbiota and pathobionts in IBD and Colo-rectal cancer

Patients with IBD have been found to have twice the concentration of mucosal *Bacteroides fragilis* biofilm compared with controls; this abnormality can in turn be corrected by antimicrobial therapy [33]. Other pathobionts implicated in the etiology of IBD include *E. coli* (eg. Adhering Invasive *E. coli*; AIEC), and *Enterococcus faecalis* [91–94]. All of these bacterial strains can produce extracellular proteases, lending further support to the hypothesis that these pathobionts may contribute to the pathogenesis of IBD via a proteolytic disruption of gut microbiota biofilms, in addition to their known detrimental effects on host tissue [88]. Indeed some *E. coli* associated with IBD contain serine protease autotransporter proteins [91], enterotoxigenic *B. fragilis* makes a zinc-containing metalloprotease enterotoxin found in IBD [92], and *E. faecalis* can produce two extracellular proteases, gelatinase and serine protease, that can induce colitis [95, 96]. The Crohn’s disease pathobiont AIEC, when given to mice as a resident microorganism, exacerbates post-infectious disease upon acute gastroenteritis caused by *Salmonella typhymurium* or *Citrobacter rodentium* [97]. Recent findings have demonstrated that microbiota biofilms from patients with Crohn’s disease or with ulcerative colitis are disrupted, and disperse pathobionts that have the ability to invade intestinal epithelia and trigger inflammation [26]. Pathobionts that have been causally implicated in the development of colo-rectal cancer also include *Bacteroides fragilis*, *E. coli*, as well as others such as *Fusobacterium nucleatum*, further linking the inflammatory processes seen in IBD with potential carcinogenesis possibly through strikingly similar microbiota-dependent pathways [80, 83, 98–101].

Healthy gut microbiota also play a key role in anti-cancer immune defense. Recent studies reported that the use of broad-spectrum antibiotics lead to unfavourable clinical outcomes in patients with various types of cancer including colo-rectal cancer [102]. Gut bacteria such as *Akkermansia muciniphila*, *Bacteroides vulgatus*, *Bifidobacterium* spp. and *Faecalibacterium* spp. (bacterial commensals also linked to protection against Inflammatory Bowel Disease [103]) have been found to play a key immuno-potentiating role in the cancer-immune dialogue, and contribute to anticancer immunity [104]. Indeed, cause-to-effect relationships between antibiotic-induced microbiota disruptions and failure of immunotherapy have been established in various cancer models treated with programmed cell death protein 1 and anti-CDLA-4 antibodies [102, 105, 106].

#### Microbiota biofilms in IBD and Colo-rectal cancer

Mechanisms regulating the biofilm mode of growth of gut microbiota in the host intestine have only begun to emerge. It was recently found that surface adhesins, Serine-Rich Repeat Proteins (SRRP’s) that bind to host epithelial proteins, were used by commensal bacteria to form their physiological biofilms in the murine gut [107]. It has now become apparent that these adhesins bind to selective features in the host in pH-dependent fashion, which may contribute to their adaptation as biofilm commensals in different niches of the gut [108]. Whether host IgA, which helps aggregate bacteria, may contribute to this phenonmenon requires further research. Regulation of virulence genes and biofilm formation in *Pseudomonas aeruginosa* is regulated by biochemical communications between bacteria within a biofilm, a process known as quorum sensing [109]. Moreover, quorum sensing modulates the social behaviour of biofilm bacteria via intracellular signaling molecules such as cyclic di-GMP, a pleiotropic second messenger that drives numerous functions in planktonic cells, and helps coordinate the transition between the planktonic and biofilm modes of growth [110]. The role of c-di-GMP in this switch has been established for a number of bacterial species, including *E. coli*, *P. aeruginosa*, and *Salmonella* sp. [111]. These and other discoveries on the mechanisms orchestrating the formation and disruptions of gut microbiota biofilms, and the dispersion of pathobionts, will help in our search towards novel therapeutic targets in a broad variety of disorders mediated by microbiota biofilm dysbiosis.

**Table:** 

Key concept Recent reports suggest that “biofilm formation” is linked to IBD, and may confer a pro-carcinogenic state [35, 40, 41]. Based on the discussion offered above, we propose that it is not the biofilm phenotype of the microbiota per se that may drive disease -the microbiota biofilm mode of growth is homeostatic-, but rather, it is its adhesion to the epithelial surface, beyond the mucus barrier, and its release of pathobionts that should be targeted for therapy. This distinction of course has significant bearing on designing avenues for future therapeutic developments.	

**Fig. 5:**
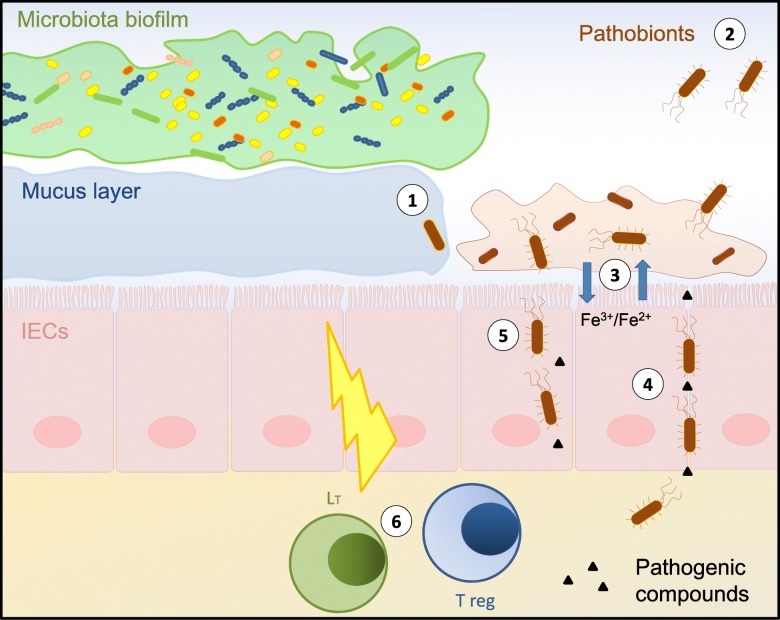
Schematic diagram illustrating the complex pathogen-commensal-mucus-tissue interactions discussed in this review. Microbiota biofilm disruption and pathobiont dispersion cause diseases resulting from microbiota dysbiosis. The data collected to support this model focus on enteropathogen-induced microbiota dysbiosis, and the microbiota disruptions reported in Inflammatory Bowel Disease and colo-rectal cancer. 1) Biofilm fragments of the microbiota cross the mucus barrier and adhere to the epithelial surface. 2) Planktonic bacteria dispersed from the microbiota biofilm may act as virulent pathobionts; adherent and motile pathobionts release pathogenic compounds (ie hemolysin, *hlyE* gene), and express genes involved in epithelial adhesion (eg *fimA*, *sfmF, fliD*). 3) Transformation of commensal microbiota bacteria into pathobionts is enabled at least in part through microbial uptake of excess iron from the intestinal environment. 4–5) Pathobionts translocate through the epithelium paracellularly [4] and transcellularly [5]. 6) Pathobionts activate host immunity to cause post-infectious and inflammatory disorders, or to exacerbate and/or cause inflammation in Inflammatory Bowel Disease, or to induce colorectal cancer

The gut microbiota biofilm disruptions seen when induced by enteropathogens, or in IBD, or in colo-rectal cancer share similarities (Fig. 5). These indicate that structural and functional microbiota biofilm dysbiosis, combined with the release of pathobionts, lie at the core of disease pathogenesis in the gut. The respective roles of each of these bacterial pathobionts, and possibly of others, as well as the mechanisms conferring virulence to them, remain obscure. The use of metagenomic and metatranscriptomic platforms will help answer these critical questions, and in turn help establish a rational basis for the development of new therapies targeting microbiota biofilm dysbiosis and pathobiont formation. Recent evidence already indicates that a patient’s microbiota plays a key role in therapeutic interventions against cancers not only in the intestine, but also at remote sites including the lungs and kidneys as well as in melanomas, paving the way towards novel approaches in precision medicine [102, 105, 106].

#### The role of iron and metabolomes of pathobionts released from microbiota biofilms

Pathogens have evolved elegant strategies to escape from commensal-induced resistance and host immunity, strategies which in turn confer the microorganisms with effective virulence factors. Production and usage of local luminal metabolites represent key regulators of pathogen-commensal-pathobiont interactions, and are critical for niche selection and for controlling infection and disease [7, 49, 112–115]. As microbiota dysbiosis is associated with changes in the levels of microbial and host metabolites, these in turn offer promise in our quest to discover novel biomarkers and therapeutic targets for microbiota dysbiosis-induced disorders.

#### The role of iron

Several species of Proteobacteria (*Enterobacteriaceae;* eg. *E. coli*, *Salmonella, Vibrio*) and Firmicutes (*Bacillus*) need to properly synthesize or incorporate metabolites, including those related to the iron-related purine and pyrimidine metabolism, from their environment to efficiently colonize and persist in the intestine, or to proliferate in the human bloodstream [116–118]. One environmental element that plays a central role in niche selection and virulence is iron. In view of recent findings on the role of iron in pathobiont release from microbiota biofilms in patients with IBD [26] this section will focus on the role of iron in these processes. Recent findings indicate that genes encoding for propanediol utilization (*pdu* operon) and iron acquisition (yersiniabactin, *chu* operon) are overexpressed in AIEC [119]. Furthermore, production of cellulose by AIEC contributes to an iron-dependent promotion of bacterial aggregation, which suggests that iron may have direct and indirect effects on biofilm formation for some species [120]. In vitro [121], in animal models [122], and in humans [123], Proteobacteria can thrive at the expense of other gut bacteria in an iron-rich environment. Thus, iron-acquisition represents a critical factor of bacterial virulence. Furthermore, pathogenic bacteria (including those from the *Enterobacteriaceae* family) are known to exhibit elevated iron uptake capacity [124], but data on human mucosal microbiota biofilms were lacking. Recent findings have now established that iron uptake is a key mechanism in conferring virulence to pathobionts dispersed by microbiota biofilms in patients with IBD [26]. Anemia is one of the most common extra-intestinal complication of IBD [125]. Intriguingly, dietary iron supplementation leads to disease exacerbation and a higher risk of infection, perhaps through alterations of commensal microbiota [126–128]. Whether intestinal bleeding associated with IBD could elevate iron concentrations in the intestinal lumen to favour populations of iron-acquiring pathobionts requires further research.

Host lipocalin 2 (Lcn2; also known as siderocalin) impairs iron acquisition by successfully competing with the iron-enterobactin uptake system in bacteria [129, 130]. This protects the host against iron-dependent bacterial pathogenesis and inflammation, including in IBD [131–133]. Lcn2, which is critical for intestinal homeostasis, is increased in the inflamed tissues of patients with IBD for unknown reasons, and is used as a biomarker of inflammation in the gut [133]. Conversely, lower Lcn2 levels in some patients with CD may reflect impaired Th17 immunity in association with the carriage of IBD-risk-increasing IL23R variants, via unclear mechanisms [133]. Knowing that iron can generate powerful radicals and that iron chelators are strong antioxidants [134] makes the clinical potential of drugs that may chelate iron in IBD even more intriguing. Whether elevated Lcn2 levels in patients with IBD is triggered by the dispersion of pathobionts with high intracellular iron has yet to be established. The significant therapeutic potential of this avenue was recently highlighted in studies that demonstrated that a new drug with potent iron-chelating properties (ATB 429; Antibe Therapeutics, Toronto, Canada) was indeed able to suppress the pathogenic effects of pathobionts that were dispersed by dysbiotic microbiota biofilms from IBD patients [26].

#### The microbiota metabolome

A number of studies have established the importance of the microbiota metabolome in health and disease. This rapidly expanding microbiota metabolome linked to human disease includes short chain fatty acids, amines, polysaccharides, primary and secondary bile acids, and various xenobiotic metabolites [135]. Particular attention has been given to bacterial short chain fatty acids, including butyrate, propionate, and acetate. These have been implicated in the modulation of inflammation, in the gut and beyond, and have been linked to the pathogenesis of IBD and cancer [136–140]. Butyrate, which is produced by metabolism of dietary fiber or unabsorbed carbohydrates in the colon, particularly by commensal bacteria belonging to the genus *Clostridia*, *Faecalibacterium*, and *Roseburia*, has been used as a prime example of how short chain fatty acids from the microbiota may regulate key physiological functions in the intestine and other organs [136–138, 140–142]. Mechanisms include the modulation of Wnt signaling, STATS-6-dependent M2 macrophage polarization, induction of the Foxp3 gene to induce Treg cells, epigenetic regulation of gene expression via miRNA, and enhancement of mitochondrial function [137, 141, 143–147]. Moreover, bacterial short chain fatty acids, including butyrate, and amino acids, may inhibit the growth of a variety of gut bacteria [115]. However, recent studies have demonstrated that early intervention with oral sodium-butyrate in neonatal piglets modulates inflammatory cytokines in the ileum, with little impact on intestinal microbiota composition [148], further highlighting the therapeutic potential of microbiota short chain fatty acids. This potential is being looked at not only for IBD and cancer, but also in a broad range of other diseases in which microbiota dysbiosis has been implicated, including autism spectrum disorders, obesity, kidney disease, and cardio-vascular diseases [140, 147, 149, 150].

In an attempt to assess microbiota metabolomics beyond the microbial secretome in fecal samples, recent analyses of microbiota biofilms grown ex vivo from biopsies of patients with IBD measured and compared the uptake (ie values lower than those of media alone) and release (ie values higher than those of media alone) of microbiota metabolites [26]. The findings indicate that iron chelating compounds increase urate release and reduce guanosine and hypoxanthine uptake in IBD biofilms, in association with the ability of these compounds to block the invasiveness and pro-inflammatoyr phenotype of the dysbiotic microbiota. These may carry physiological significance as elevated serum levels of urate have been associated with chronic inflammatory and metabolic diseases (hypertension atherosclerosis, diabetes), possibly through urate’s pro- and anti-oxidative properties and its interactions with iron [151]. Hypoxanthine, a purine derivative, is used for DNA synthesis as a nutrient by various bacteria, including *E. coli* and *Enterobacteria cloacae* [152, 153]. Whether elevated uptake of hypoxanthine by dysbiotic microbiota may contribute to the proliferation of pathogenic *Enterobacteriaceae* in IBD and in other diseases is unknown.

More research is needed to establish cause-to-effect relationships between microbiota phenotype/function, the microbiota exometabolome, and disease.

## Conclusion

A plethora of conditions have been linked to disruption of the gut microbiome. Mechanisms remain incompletely understood, but hold the promise to reveal a path towards new disease markers and therapies. The gut microbiota live in a biofilm mode of growth. The processes promoting this biofilm phenotype in the gut have only begun to emerge. Surface adhesins such as Serine-Rich Repeat Proteins are used by commensal bacteria to form biofilms and for niche selection along the gut. Bacteria from the gut microbiota are enriched with genes linked to biofilm formation, encoding for type IV pili, motility, flagella, intracellular trafficking, adhesins, and biofilm-associated proteins. Biochemical communications between bacteria within the biofilm as well as between planktonic cells, a process known as quorum sensing, modulates the social behaviour of biofilm bacteria via signaling molecules such as cyclic di-GMP, which coordinates some of the steps in the transition between the planktonic and biofilm modes of growth. This review highlights recently discovered pathogenic mechanisms that appear to be shared when induced by enteropathogens, Inflammatory Bowel Disease, and colo-rectal cancer. Beyond the classical disruptions of microbial abundance and diversity, these shared processes involve the disturbance of the gut microbiota biofilm phenotyoe and function, and the dispersion of planktonic bacteria from these commensal communities. These dispersed microorganisms may become pathobionts, and have been causally implicated in disease development, at least in part by invading the mucus barrier, adhering the intestinal epithelium, translocating through enterocytes, and activating various pro-inflammatory pathways, including a recently uncovered mechanism that promotes regulatory T cells which suppress protective responses to inflammatory stimuli. In IBD and colo-rectal cancer, the best characterized bacterial commensals escaping from a disturbed microbiota biofilm and turned pathobionts include Adhering Invasive *E. coli* (AIEC)*, Bacteroides fragilis*, *Enterococcus faecalis*, and *Fusobacterium nucleatum.* Iron-acquisition is a critical factor for bacterial pathogenicity, and recent discoveries have linked this process to the expression of virulence factors in gut commensals that have become pathobionts. More research in the metagenomic, metatranscriptomic, and metabolomic (both for the uptake and release of metabolites) profiles of pathobionts dispersed from dysbiotic gut microbiota biofilms will pave the way towards the developments of new therapies that may restore homeostasis. In view of the numerous clinical conditions that result from microbiota dysbiosis, this field of microbiome research carries enormous translational potential.
